# Efficiency improvement by using metal–insulator-semiconductor structure in InGaN/GaN micro-light-emitting diodes

**DOI:** 10.1007/s12200-024-00111-9

**Published:** 2024-03-28

**Authors:** Jian Yin, David Hwang, Hossein Zamani Siboni, Ehsanollah Fathi, Reza Chaji, Dayan Ban

**Affiliations:** 1 Department of Electrical and Computer Engineering, Waterloo Institute Nanotechnology, University of Waterloo, Waterloo, ON N2L 3G1 Canada; 2 Vuereal InC., 440 Philip Street, Unit 100, Waterloo, ON N2L 5R9 Canada

**Keywords:** Micro-LED, GaN, EQE improvement, Micro-fabrication

## Abstract

InGaN/GaN micro-light-emitting diodes (micro-LEDs) with a metal–insulator-semiconductor (MIS) structure on the sidewall are proposed to improve efficiency. In this MIS structure, a sidewall electrode is deposited on the insulating layer-coated sidewall of the device mesa between a cathode on the bottom and an anode on the top. Electroluminescence (EL) measurements of fabricated devices with a mesa diameter of 10 μm show that the application of negative biases on the sidewall electrode can increase the device external quantum efficiency (EQE). In contrast, the application of positive biases can decrease the EQE. The band structure analysis reveals that the EQE is impacted because the application of sidewall electric fields manipulates the local surface electron density along the mesa sidewall and thus controls surface Shockley–Read–Hall (SRH) recombination. Two suggested strategies, reducing insulator layer thickness and exploring alternative materials, can be implemented to further improve the EQE of MIS micro-LEDs in future fabrication.

**Graphical Abstract:**

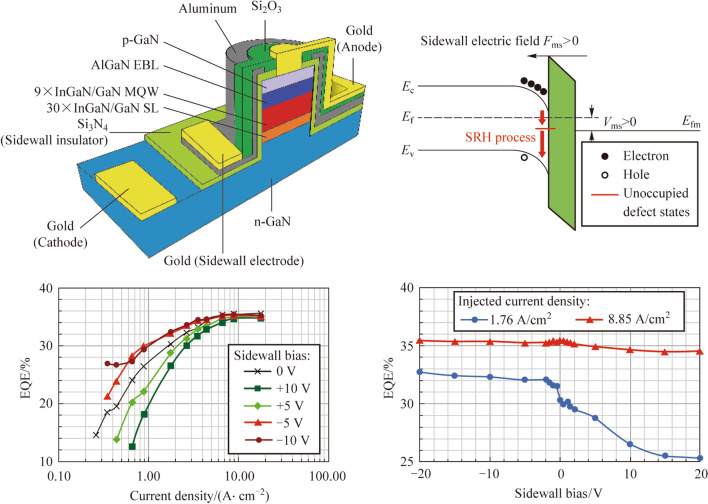

## Introduction

Ever since the demonstration of the InGaN/GaN blue light-emitting diode (LED), it has become valued as the light source in different applications in the past 30 years. Compared to other candidate sources, the InGaN/GaN blue LED has the highest brightness and longest lifetime. Therefore, it is widely used in various displays on monitors, cellphones, and televisions. With the growing demand for ultra-high resolution displays, size reduction of InGaN/GaN blue LEDs is essential. Previous studies have revealed a trend of reduced external quantum efficiency (EQE) with smaller LEDs, and this is the key factor in limiting the application of InGaN/GaN micro-LEDs [1–6]. The EQE decrease of InGaN/GaN micro-LEDs is due to the significant surface recombination via the surface defects along the sidewall resulting from the plasma-assisted dry etching process [7, 8]. To suppress the surface recombination and improve the EQE of InGaN/GaN micro-LEDs, some methods have been reported in recent years, such as sidewall passivation and annealing [8–13]. However, the improvement of EQE was not enough to meet the requirement for the new generation displays.

In this paper, an InGaN/GaN micro-LED with a metal–insulator-semiconductor (MIS) structure on the sidewall is proposed to achieve a high efficiency InGaN/GaN micro-LED. Metal is deposited on the passivated insulator along the sidewall as the sidewall electrode. Electroluminescence (EL) measurements show that the EQE values of MIS micro-LEDs with a mesa diameter of 10 μm increase with negative biases and decrease with positive biases applied to the sidewall electrode. The theoretical analysis of the band structure of the sidewall explains how the sidewall biases affect the carrier concentration and recombination on its surface. The efficiency improvement and variation of current–voltage (*I–V*) characteristics of micro-LEDs with MIS structures are attributed to the application of sidewall electric fields. The simulated Shockley–Read–Hall (SRH) recombination distribution along the quantum wells region under different sidewall biases supports the conclusion obtained from band structure analysis. To further improve the efficiency of MIS micro-LEDs in future fabrication, reducing the passivated insulator thickness and applying high-*κ* materials are two possible strategies to increase the sidewall electric field.

## Experimental setup

**Fig. 1:**
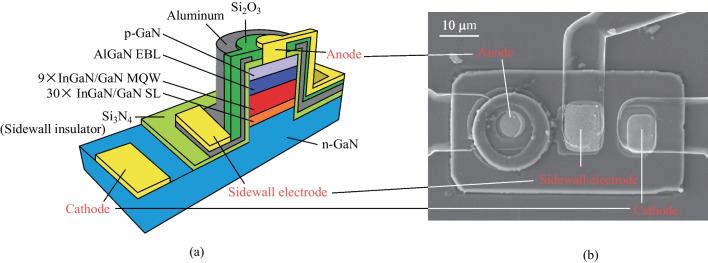
**a** Cross-section schematic of the MIS micro-LED design and **b** top-view SEM image of the MIS_10. The lines linking **a** and **b** match the positions of three electrodes in the two images

The InGaN/GaN micro-LED with MIS structure under investigation was grown on a *c*-plane planar sapphire substrate with mesa diameters of around 10 μm (denoted herein as devices MIS_10). The epitaxial structures of the micro-LED were grown by metal–organic chemical vapor deposition (MOCVD), which consist of a 2 μm Si-doped n-GaN layer, a 0.3 μm superlattice layer, 9 periods of InGaN/GaN multiple quantum wells (MQWs) and slightly p-doped quantum barriers (3 nm/12 nm), a 30 nm Mg-doped p-AlGaN electron blocking layer, and a 40 nm Mg-doped p-GaN layer. Piranha and solvent cleaning procedure were carried out to remove the potential contaminations before the initial fabrication process. Following the cleaning steps, we defined circular mesas with 10 μm diameters by reactive-ion etching (RIE) to etch down to the n-GaN layer using chlorine/boron trichloride/argon. The mesas were subsequently treated with potassium hydroxide (KOH), buffered hydrofluoric acid (BHF), and piranha solution at room temperature for sidewall treatment. After sidewall treatment, a 600 nm silicon nitride (Si_3_N_4_) layer was deposited to form the sidewall insulator layer in the MIS structure by using plasma-enhanced chemical vapor deposition (PECVD). The selective areas of Si_3_N_4_ were removed by using BHF for ohmic contact windows. The p- and n-contacts were composed of 10/10 nm of Ni/Au and 30/10 nm of Cr/Au, respectively, through e-beam evaporation. Following the deposition of these contacts, a 150 nm Aluminum (Al) layer was deposited as the sidewall metal layer in the MIS structure by using sputter deposition. The sidewall electrode was defined by RIE and then isolated by a 200 nm SiO_2_ layer. The selective areas of SiO_2_ were removed by using RIE for metal electrode windows. The three electrodes (anode, cathode and sidewall electrode) were coated with 750 nm of Au through e-beam evaporation and were patterned via wet etching. The cross-section schematic and top-view SEM image of the MIS_10 are shown in Fig. 1. In the final step of the fabrication process, the micro-LED wafer was diced into 1 cm × 1 cm chips. The diced chips were then mounted onto the designed printed circuit board (PCB) and wire-bonded. The *I–V* characteristics and EQE values of MIS_10 were measured using a Keithley 2400 source meter unit (SMU) and an integrating sphere at room temperature. The cathode of MIS_10 was connected to the ground of the SMU.

## Results and discussion

**Fig. 2:**
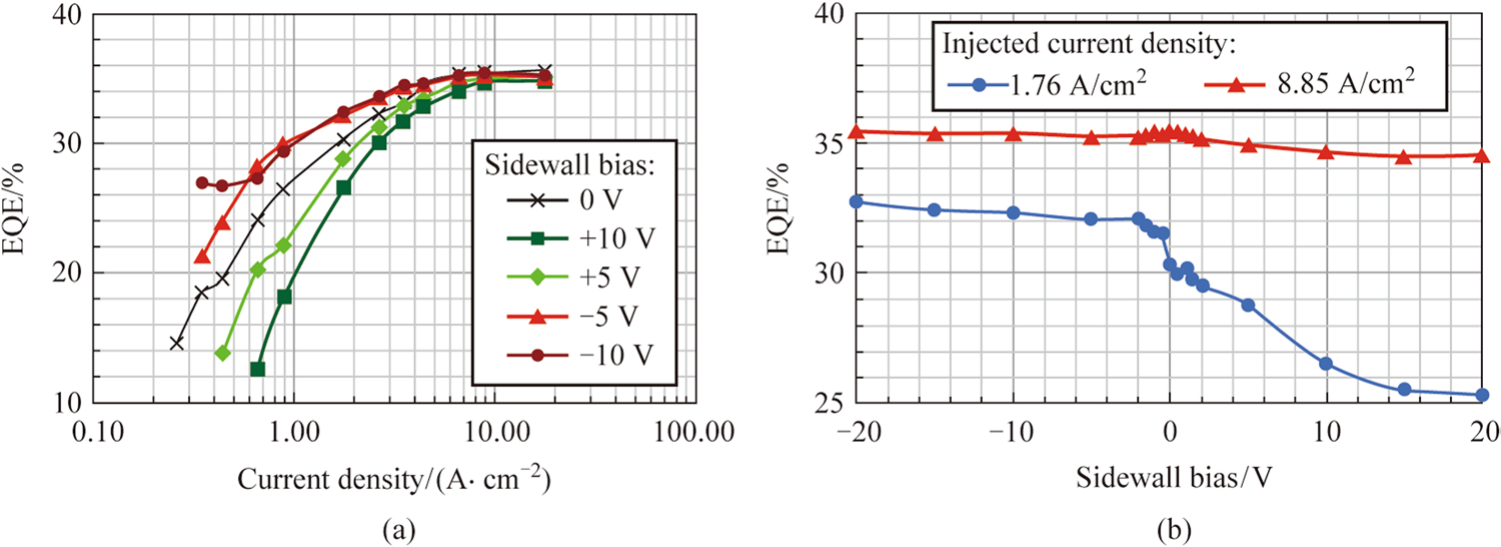
**a** EQE values as functions of injected current densities for MIS_10 with different sidewall biases. **b** EQE values as functions of sidewall bias for MIS_10 with different injected current densities

Figure 2 describes the EQE performance of MIS_10 with different biases applied to the sidewall electrodes. Figure 2a exhibits the EQE values of MIS_10 as functions of injected current densities with different sidewall biases. Applying negative biases to the sidewall electrode can improve the efficiency at the low current injection region (< 8 A/cm^2^). In contrast, positive biases can decrease the EQE at the low current injection region. At 1.76 A/cm^2^, applying − 10 V can improve the EQE from 30.30% to 32.29% and applying + 10 V can decrease the EQE from 30.30% to 26.48% (as shown in Fig. 2b). When the injected current density is higher than 8 A/cm^2^, the increase or decrease of EQE with different sidewall biases becomes negligible. Figure 2b shows that at 8.85 A/cm^2^, the EQE variation of MIS_10 is less than 1% with different sidewall biases (from − 20 to + 20 V).

**Fig. 3:**
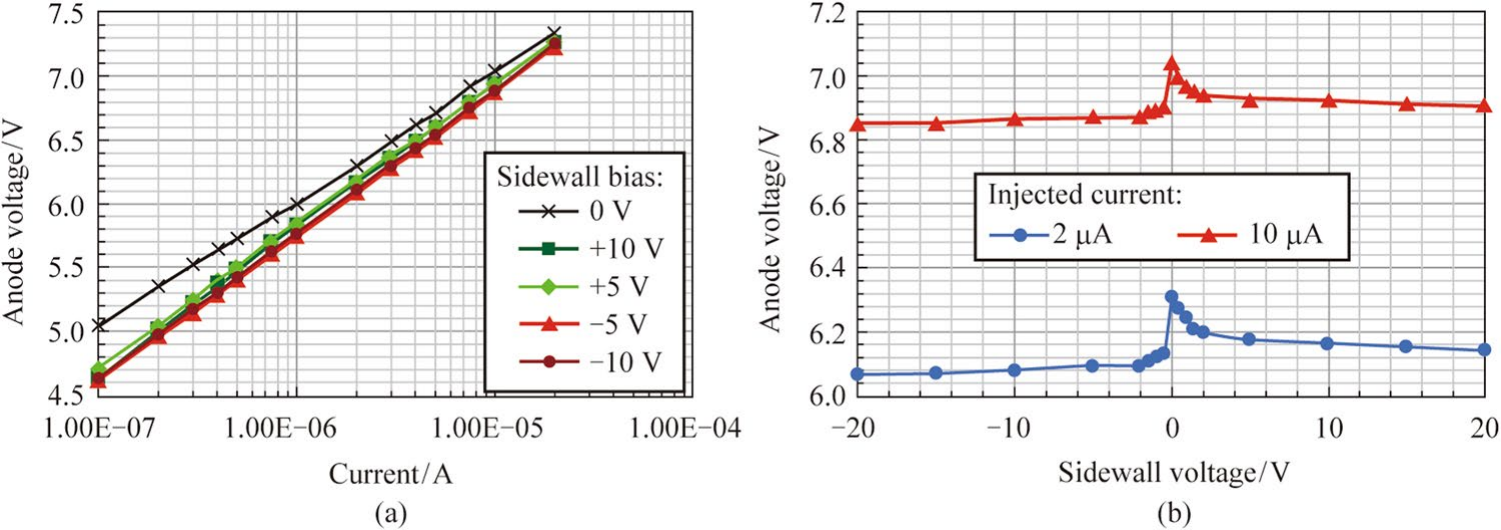
**a** Anode voltages as functions of injected currents for MIS_10 with different sidewall biases. **b** Anode voltages as functions of the sidewall biases for MIS_10 with different injected currents

Figure 3 shows the* I–V* characteristic of MIS_10 with different biases applied to sidewall electrodes. In Fig. 3a, the applied voltages on the anode are presented as functions of the injected currents for MIS_10 with different sidewall biases. At the same injected current, applying voltages to the sidewall electrode (whether positive or negative) causes a slight reduction in the anode voltage. For instance, at an injected current of 2 μA, applying − 10 V lowers the anode voltage from 6.31 to 6.08 V, while applying + 10 V reduces it to 6.16 V (as illustrated in Fig. 3b). As the injected current increases, the discrepancies among the various *I–V* curves with different sidewall biases decrease. The unusual high anode voltage observed in MIS_10 is attributed to the poor ohmic contact between the p-GaN layer and the metal layer (Ni/Au).

**Fig. 4:**
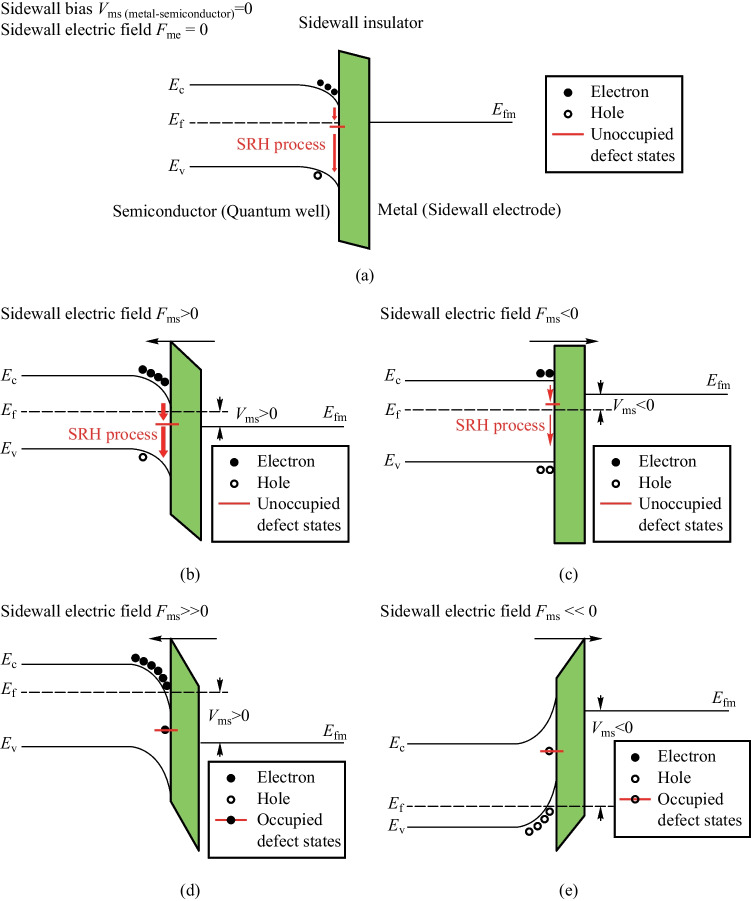
Band structure diagrams of the sidewalls for quantum wells in MIS_10 with different sidewall electric fields: **a** *F*_ms (metal–semiconductor)_ = 0, **b** *F*_ms_ > 0, **c** *F*_ms_ < 0, **d** *F*_ms_ $\gg$ 0, and **e** *F*_ms_ $\ll$ 0

To explain the underlying mechanisms of MIS micro-LEDs, Fig. 4 shows the band structure of MIS structure on the sidewall in MIS_10 with different sidewall biases and different sidewall electric fields. Figure 4a represents accumulation of electrons on the semiconductor/sidewall-insulator interface without sidewall biases. One of the reasons for the accumulation of electrons at the semiconductor/insulator interface is the presence of slightly p-doped quantum barriers within the active region of the device. Moreover, in p-type semiconductors, we are particularly concerned with the lifetime and diffusivity of minority carriers, which, in this case, are electrons. The other reason is the mobility imbalance between electrons and holes in InGaN and GaN. Some injected electrons, with higher mobility than holes, pass through the active region and recombine with the injected holes in p-GaN. This results in a higher injected non-equilibrium electron density than injected non-equilibrium hole density in the active region, leading to the accumulation of injected electrons at the sidewall interface. The accumulated electrons on the semiconductor/sidewall-insulator interface will be captured by the acceptor traps (electron traps) on the sidewall surface and jump to the valence band, which is depicted as surface SRH recombination process (shown in Fig. 4a). The surface SRH recombination constitutes the leakage current along the sidewall and results in the lowered efficiency of micro-LEDs. When the positive electric field *F*_ms_ is applied (Fig. 4b), more electrons are accumulated on the sidewall interface, resulting in higher surface SRH recombination. In contrast, when the negative electric field *F*_ms_ is applied (Fig. 4c), fewer electrons accumulate on the sidewall interface, resulting in lower surface SRH recombination. While a negative electric field does attract the holes accumulated on the sidewall interface, it’s important to note that, in devices with slightly p-doped quantum barriers, the majority carriers (holes) do not significantly influence the surface recombination [14, 15]. Figures 4b and c explain the sidewall bias-dependent EQE of MIS_10 at the low current injection region (i.e., at 1.76 A/cm^2^ as shown in Fig. 2b). For MIS_10 with higher current injection, the electrons repelled or attracted by the sidewall bias are only a small fraction of the injected electrons. Therefore, the increase or decrease of EQE becomes smaller at the high current injection region (i.e., at 8.85 A/cm^2^ as shown in Fig. 2b). On the other hand, the electric field applied to the sidewall reduces the mobility of carriers near the sidewall surface. This effect can be considered as due to a resistance operating in parallel with the micro-LED device [16]. The presence of this parallel resistance causes a reduction in the applied voltage at the anode under the same injected current conditions. This phenomenon accounts for the observed variation in *I–V* characteristics with sidewall biases, as illustrated in Fig. 3.

In addition, the theoretical analysis shows that even much higher positive or negative electric fields may provide better efficiency improvement. For instance, in Fig. 4d, an ultra-high positive electric field repels most holes from the sidewall. The Fermi level is higher than the conduction band near the sidewall surface. In this condition, most acceptor traps are occupied, leading to a very low surface SRH recombination rate. On the other hand, most electrons are repelled from the sidewall by an ultra-high negative electric field with the Fermi level lower than the valence band near the sidewall surface (as shown in Fig. 4e). There is almost no electron accumulation on the sidewall interface, culminating in a minimal surface SRH recombination rate. Therefore, no matter whether positive or negative, an ultra-high sidewall electric field should significantly improve the efficiency of MIS micro-LEDs.

To verify the conclusion from band structure analysis, the simulation was conducted by using the commercial simulation tool Apsys (Advanced Semiconductor Device Simulator) provided by Crosslight Software. To calculate the optical and electrical properties of the InGaN/GaN MQW micro-LEDs, the simulation tool Apsys has various models. For carrier transport calculation in the semiconductor, the drift diffusion model based on the semiconductor transport equation was used in the simulation. To analyze the optical properties of the micro-LEDs, the ABC model that included non-radiative and radiative recombination isemployed in Apsys. The ABC model can be described as

1 $${\eta }_{{\text{IQE}}}=\frac{{\eta }_{{\text{inj}}}B{n}^{2}}{An+B{n}^{2}+C{n}^{3}},$$

where ${\eta }_{{\text{IQE}}}$ represents the internal quantum efficiency (IQE), ${\eta }_{{\text{inj}}}$ denotes the injection efficiency (the ratio of carriers that recombine in the MQW region), *n* stands for the carrier density, *A* represents the SRH coefficient, *B* denotes the radiative coefficient, and *C* represents the Auger coefficient [17]. The injection efficiency, ${\eta }_{{\text{inj}}}$, depends on the built-in polarization and the electron blocking layer conduction band offset ratio $\Delta {E}_{{\text{c}}}/\Delta {E}_{{\text{g}}}$. In this simulation, the polarization factor was assumed to be 50%, and the $\Delta {E}_{{\text{c}}}/\Delta {E}_{{\text{g}}}$ was set as 0.7/0.3. The bulk SRH lifetime, Auger coefficient, and surface recombination velocity were assumed to be 100 ns, 1 × 10^−30^ cm^6^/s, and 1 × 10^4^ cm/s [15], respectively. To accurately describe the surface recombination model in the sidewall region, the capture cross sections of traps were set at 3.4 × 10^−17^ and 2.1 × 10^−15^ cm^2^ for electrons and holes, respectively [18–20]. The distribution of electron traps was uniform from 0.8 eV below the conduction band to the conduction band and was assumed to be 1 × 10^12^ cm^−2^⋅eV^−1^, while the distribution of hole traps exponentially decreased from the valence band to 1.5 eV below the valence band and was set as ${10}^{12}{\text{exp}}\left({E}_{{\text{v}}}-E/0.11\right){{\text{cm}}}^{-2}{\cdot {\text{eV}}}^{-1}$ [21].

**Fig. 5:**
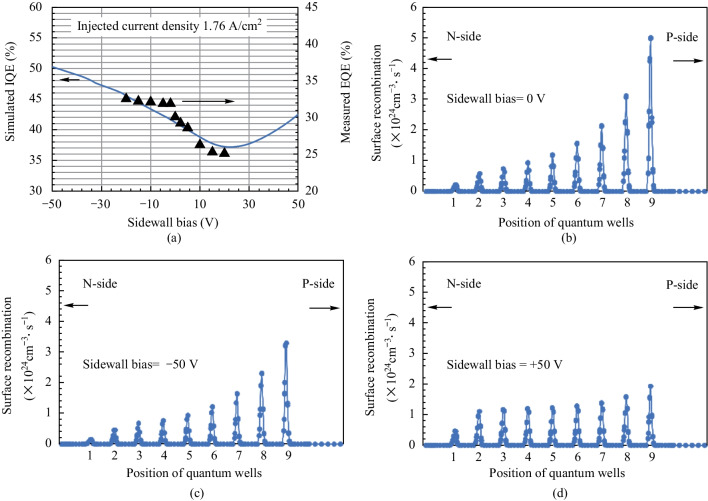
Simulation results: **a** simulated IQE values (blue line) as functions of sidewall biases at 1.76 A/cm^2^ for MIS_10. The triangle marks are the measured EQE data from Fig. 2; **b**–**d** represent the simulated surface recombination distribution across the multiple quantum wells of MIS_10 with different sidewall biases **b** 0 V, **c** − 50 V, and **d **+ 50 V

Figure 5a illustrates the simulated IQE and measured EQE (from Fig. 2b) as functions of sidewall biases at 1.76 A/cm^2^. With a consistent sidewall bias-independent light extraction efficiency, the simulated IQE matched the measured EQE values of MIS_10. As the sidewall bias decreased from 0 to − 50 V, the IQE values of MIS_10 increased from 41.2% to 50.2%. In contrast, with an increase in sidewall bias from 0 to + 50 V, the IQE reached a minimum at a certain positive sidewall bias (around + 25 V) and then increased. Figures 5b–d exhibit the surface SRH recombination distribution along the quantum wells region of MIS_10 under different sidewall biases. Since the mobility of electrons in GaN is much higher than the hole mobility, most of the recombination occurred near the p-region. Therefore, the surface recombination rate at the 9th quantum well was the highest, as depicted in Fig. 5b. As applying a sidewall bias of − 50 V, Fig. 5c shows a consistent decrease in surface recombination rates across various quantum wells. The maximum surface recombination rate at the 9th quantum well decreased from 5.01 × 10^24^ to 3.46 × 10^24^ cm^−3^·s^−1^, and the integrated surface recombination rate along multiple quantum wells decreased from 2.48 × 10^18^ to 1.77 × 10^18^ cm^−2^·s^−1^. In this condition, most electrons were repelled from the sidewall by high negative electric field, which results in reduced surface recombination. On the other hand, Fig. 5d indicates a more balanced distribution of surface recombination rates along quantum wells when a sidewall bias is + 50 V. The maximum surface recombination rate at the 9th quantum well became 1.94 × 10^24^ cm^−3^·s^−1^, and the integrated surface recombination rate along the quantum wells was 1.97 × 10^18^ cm^−2^·s^−1^. This value was higher than that with a sidewall bias of − 50 V but lower than that with a 0 V sidewall bias. With high positive electric field, the electrons are attracted to the sidewall and occupy most of the acceptor traps, which results in lower surface recombination. This condition lowers the speed of electrons, and balances the mobility between electrons and holes. Therefore, the surface recombination rates at various quantum wells are getting closer to each other.

However, for MIS_10, the biases and electric fields required to reach the conditions shown in Fig. 4d and e are exceedingly high. With a 600 nm insulator in our MIS device, the sidewall biases need to be in the tens or even hundreds of volts range. Thus, to attain the high electric field condition depicted in Figs. 4d and e, there are two potential methods for future improvement: reducing the thickness of the sidewall insulator and increasing the permittivity of the insulator layer. For the former method, a better deposition method is required. Compared to PECVD, atomic layer deposition (ALD) is a better deposition method with higher resolution [8–10, 22–25]. Due to the one-molecule-layer deposition per cycle process, ALD-passivated layers exhibit superior conformal coverage, resulting in more substantial suppression of surface recombination compared to PECVD-passivated layers. When the insulator thickness is less than 60 nm, ALD can deposit a higher-quality insulator in MIS structures. However, for insulator thicknesses exceeding 60 nm, which surpass the critical deposition thickness of ALD, a combined ALD/PECVD passivation still offers better suppression of surface recombination than single PECVD passivation [18]. For the latter, high-*κ* dielectrics are preferred to reach the high electric field with a lower sidewall bias. Common materials to replace Si_3_N_4_ as sidewall insulators can be Al_2_O_3_, HfO_2_ and ZrO_2_ [8–10, 22–26].

## Conclusions

In summary, InGaN/GaN micro-LEDs with MIS structures are proposed to improve efficiency by depositing metal on the sidewall as a sidewall electrode. The measured EQE values of the device with a 10 μm diameter mesa indicate that applying negative or positive biases on the sidewall electrode increase or decrease the EQE, respectively. The underlying mechanisms of micro-LEDs with MIS structures on the sidewall are investigated by analyzing the band structures of MIS structures. Negative (positive) biases on the sidewall electrode can repel (attract) the electrons and thus increase (decrease) the surface SRH recombination on the sidewall, which explains the sidewall bias-dependent efficiency performance of MIS micro-LEDs. The variation in *I–V* characteristics under different sidewall biases can be attributed to the reduction in carrier mobility due to the applied electric field on the sidewall. The band structure analysis also indicates that high sidewall electric fields can suppress most surface SRH recombination and significantly improve the efficiency. The simulated SRH recombination distribution along the quantum wells region under different sidewall biases further corroborates the theory from band structure analysis. Therefore, a thinner sidewall insulator layer with high-κ dielectric can be adopted to further improve the device performance of InGaN/GaN micro-LEDs with MIS structures.

## Data Availability

Data underlying the results presented in this paper are not publicly available at this time but may be obtained from the authors upon reasonable request.
