# Correction: Efficiency improvement by using metal–insulator-semiconductor structure in InGaN/GaN micro-light-emitting diodes

**DOI:** 10.1007/s12200-024-00114-6

**Published:** 2024-04-12

**Authors:** Jian Yin, David Hwang, Hossein Zamani Siboni, Ehsanollah Fathi, Reza Chaji, Dayan Ban

**Affiliations:** 1 Department of Electrical and Computer Engineering, Waterloo Institute Nanotechnology, University of Waterloo, Waterloo, ON N2L 3G1 Canada; 2 Vuereal InC, 440 Philip Street, Unit 100, Waterloo, ON N2L 5R9 Canada

**Correction** **:** **Front. Optoelectron. 17, 8 (2024)**

**https://doi.org/10.1007/s12200-024-00111-9**

Following publication of the original article [1], the authors reported errors in the legend of Graphical Abstract, Figure 2 and Figure 3.

The Graphical Abstract has been updated from:


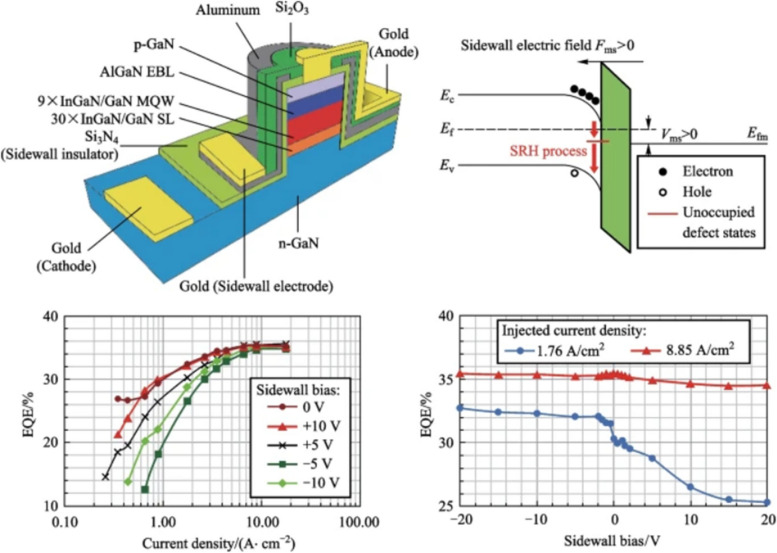


To:


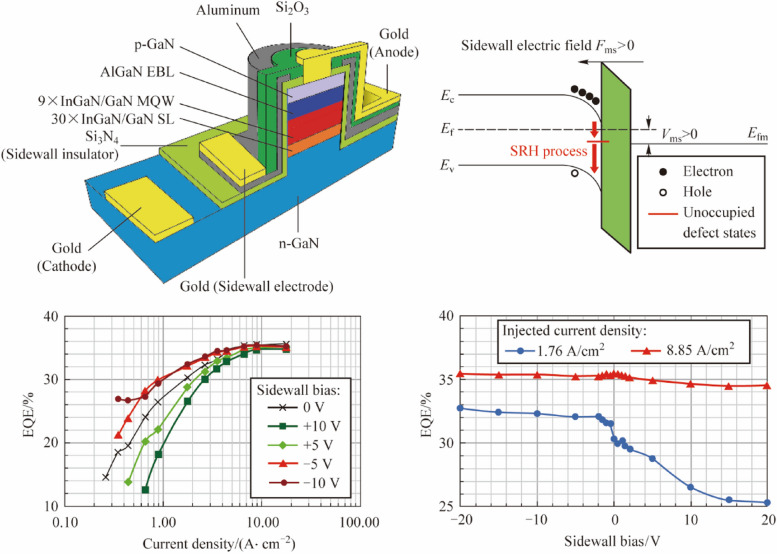


Figure 2 has been updated from:


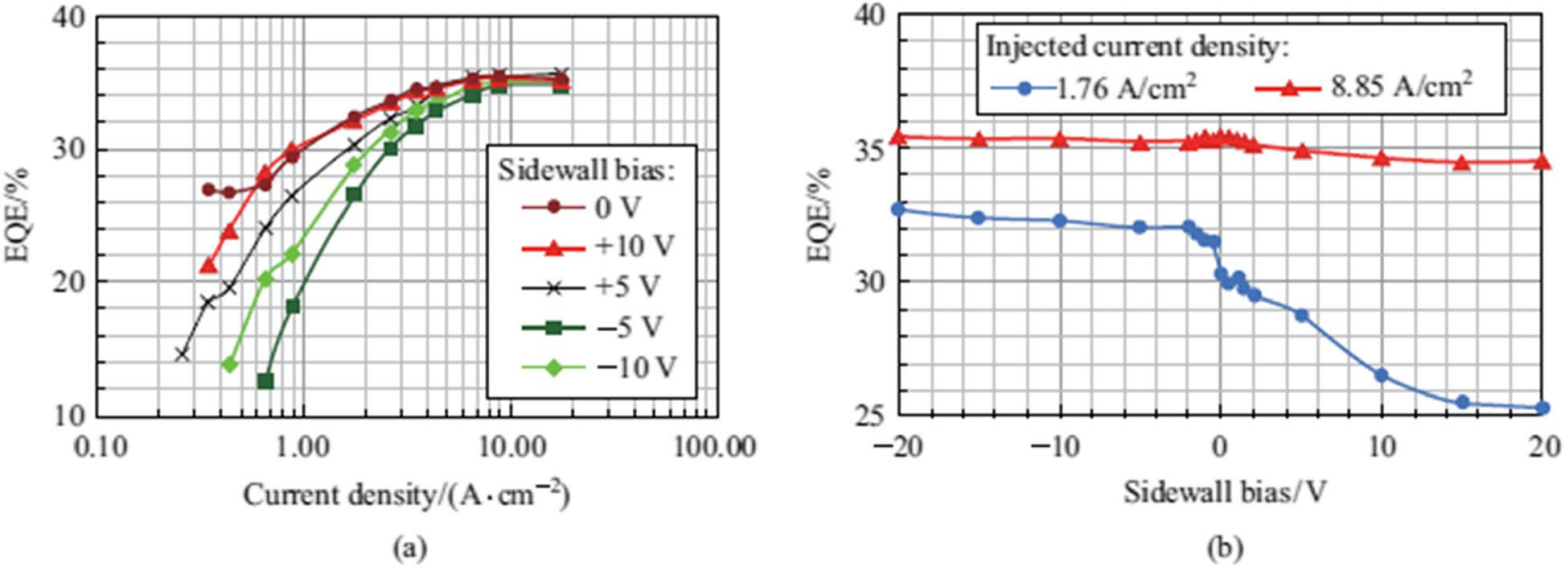


To:


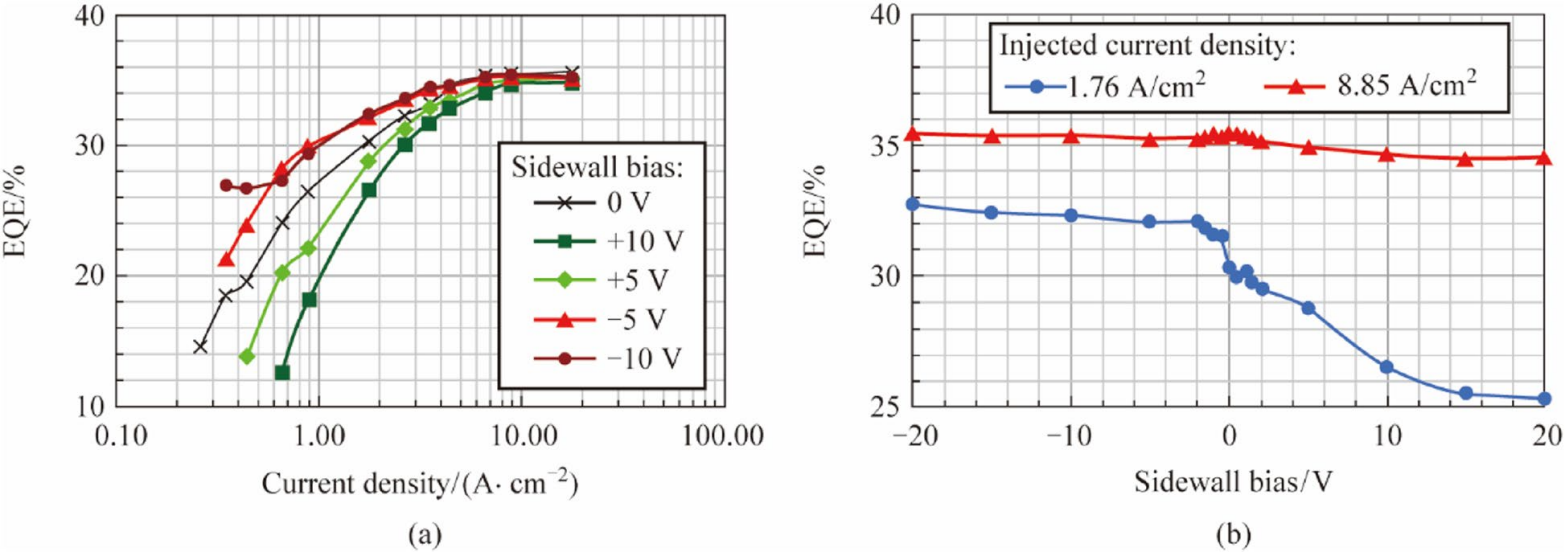


Figure 3 has been updated from:


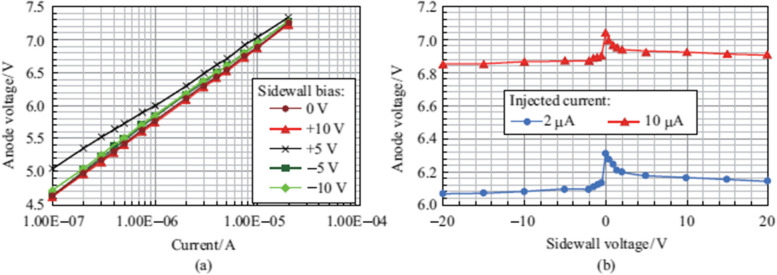


To:


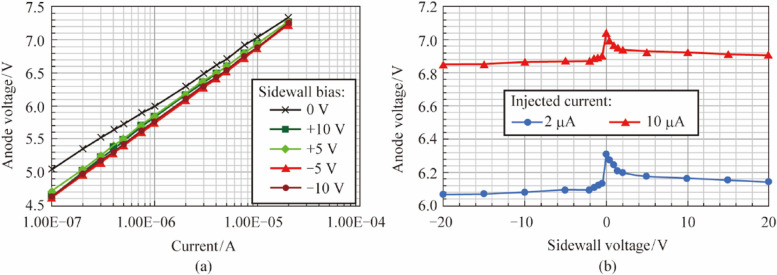


The original article [1] has been updated.
